# A pilot study evaluating alternative approaches of academic detailing in rural family practice clinics

**DOI:** 10.1186/1471-2296-13-129

**Published:** 2012-12-31

**Authors:** Daniel M Hartung, Ann Hamer, Luke Middleton, Dean Haxby, Lyle J Fagnan

**Affiliations:** 1 Oregon State University College of Pharmacy, Oregon Health & Science University, 3303 SW Bond Ave; CH12C, Portland, OR, 97239, USA; 2 Oregon Health & Science University, Oregon Rural Practice-based Research Network, 3181 SW Sam Jackson Park Rd, Mail Code: L222, Portland, OR, 97239, USA; 3 Alternative Methods for Disseminating Evidence-based Prescription Drug Information among Primary Care Clinicians in Rural Oregon: The Rural Oregon Academic Detailing Project (ROAD). American College of Clinical Pharmacy Annual Meeting, Pittsburgh, PA, USA

## Abstract

**Background:**

Academic detailing is an interactive, convenient, and user-friendly approach to delivering non-commercial education to healthcare clinicians. While evidence suggests academic detailing is associated with improvements in prescribing behavior, uncertainty exists about generalizability and scalability in diverse settings. Our study evaluates different models of delivering academic detailing in a rural family medicine setting.

**Methods:**

We conducted a pilot project to assess the feasibility, effectiveness, and satisfaction with academic detailing delivered face-to-face as compared to a modified approach using distance-learning technology. The recipients were four family medicine clinics within the Oregon Rural Practice-based Research Network (ORPRN). Two clinics were allocated to receive face-to-face detailing and two received outreach through video conferencing or asynchronous web-based outreach. Surveys at midpoint and completion were used to assess effectiveness and satisfaction.

**Results:**

Each clinic received four outreach visits over an eight month period. Topics included treatment-resistant depression, management of atypical antipsychotics, drugs for insomnia, and benzodiazepine tapering. Overall, 90% of participating clinicians were satisfied with the program. Respondents who received in person detailing reported a higher likelihood of changing their behavior compared to respondents in the distance detailing group for five of seven content areas. While 90%-100% of respondents indicated they would continue to participate if the program were continued, the likelihood of participation declined if only distance approaches were offered.

**Conclusions:**

We found strong support and satisfaction for the program among participating clinicians. Participants favored in-person approaches to distance interactions. Future efforts will be directed at quantitative methods for evaluating the economic and clinical effectiveness of detailing in rural family practice settings.

## Background

Prescribing quality is widely acknowledged to be highly variable and suboptimal according to a multitude of indicators [1,2]. The growing costs of healthcare make these deficiencies even more glaring.

Academic detailing, a term coined by Jerry Avorn, MD in the mid 1980s, is an approach aimed at improving prescribing practices and other medical decision-making using proactive outreach with non-commercial, evidence-based medical information in a user friendly format [3]. The academic detailing model is based largely on successful strategies developed and refined by pharmaceutical industry marketing departments. Detailers, who are commonly clinical pharmacists, nurses, or physicians, are trained to employ a variety of the same social marketing strategies as their industry counterparts. In a seminal paper on the topic, Soumerai and Avorn summarized the basic elements critical for a successful program as: a focused problem or objective, understanding target audience motivations, establishing credibility, encouraging two-sided communication and promotion of an active learning environment, repetition and reinforcement, and the use of brief graphical printed material [4].

There is a large and growing literature demonstrating the effectiveness of academic detailing. Several randomized trials of academic detailing have documented significant improvements in prescribing practices in areas ranging from the use of psychoactive drugs in the nursing home to rational antibiotic use [3,5]. A Cochrane Systematic Review of 69 randomized trials of educational outreach visits (e.g., academic detailing) found that this approach is associated with significant improvements in targeted behavior changes [6]. Quantitative meta-analyses from this review suggest academic detailing is associated with a 5.6% absolute improvement in the targeted behavior. As one might expect, considerable heterogeneity exists in the topics and general format of the programs studied. Some outreach interventions were multifaceted and coupled with other interventions such as audit reports. In this review, multifaceted interventions were associated with a larger effect size (8.8% absolute difference in behavior), but the change was not statistically different from the overall effect.

The reach and influence of the pharmaceutical industry on clinical practice has been documented for over 50 years [7-9]. Interaction with industry is particularly high among family practitioners relative to other medical specialties [7]. As clinicians become more acutely aware of the problems associated with industry-related conflict of interest, academic detailing may emerge as an appealing alternative [10]. Moreover, academic detailing programs are considered by some to an attractive strategy in which to bridge the evidence translation gap [11].

The need to effectively communicate new clinical research is magnified by the substantial recent investments in patient-centered comparative effectiveness research [12].

The economic implications of academic detailing programs are mixed, and reflect component costs and effectiveness of both the behavior change and the detailing program itself [13-16]. In-person outreach may not be economically feasible in geographically remote or rural areas and is a particular concern. Using technology to more efficiently deliver education to these distant regions may be a solution. The feasibility of employing technology to augment academic detailing efforts has not been formally studied or described. The overarching objective of this project was to develop and gain experience using a distance-learning academic detailing model. Specifically, the goal of this pilot study was to gain insight on the effectiveness and satisfaction of such a program in family physicians practicing in rural Oregon.

## Methods

The Rural Oregon Academic Detailing (ROAD) project was a collaboration between the Oregon State University College of Pharmacy and the Oregon Rural Practice-based Research Network (ORPRN) at Oregon Health & Science University (OHSU). ORPRN is an Agency for Healthcare Research and Quality (AHRQ) recognized Practice-based Research Network consisting of 46 primary care clinics in rural Oregon who participate in community-based research with the goal of improving the health of rural populations. Four family practice clinics were recruited to participate based on geographic area, clinic interest, and past experience with other ORPRN projects.

Following recruitment, we conducted a focus group with each clinic to assess baseline attitudes and preferences about prescription drug information and collect logistic data relevant to delivery of education (e.g., what times and dates would work for scheduling, contact information). During the focus groups, the ROAD outreach team (DH, AH) met in person with clinicians at each clinic over a period of 2 months to lay out an overview of the project, describe the premise and goals of academic detailing, outline what would be expected of the clinic, discuss what therapeutic areas each clinic would find most helpful, and, finally, to explore which educational formats would be most beneficial. Each clinic was given a $1000 honorarium for participating in the focus group and survey work. Because the State of Oregon Medicaid program co-funded this project, clinics were encouraged to select educational topics of highest relevance to the state such as management of mental illness. Specific topics suggested included mental health pharmacotherapy such as treatment of depression, use of antipsychotics in primary care, anxiety disorders, bipolar disorder, insomnia, and the use of anticonvulsants for mental illness. Based on these initial discussions, ROAD project leadership assigned two clinics to a face-to-face educational approach and two clinics to a mix of technology-enabled distance detailing. These decisions were made based on each clinic’s comfort and capacity to receive distance technology.

The education delivery component was divided into two distinct outreach periods separated by a midpoint break for survey work and approach modification if necessary. For each outreach period our detailer engaged with clinics two times about topics related to the treatment of mental illness. For the first period, we developed and delivered education focused on therapeutic choices for depression following initial antidepressant failure. The initial module in this phase drew content heavily from the National Institutes of Mental Health-funded Sequenced Treatment Alternatives to Relieve Depression (STARD) trial [17]. We also developed material summarizing the use and monitoring of atypical antipsychotics for resistant depression in a family medicine setting. The second period modules covered pharmacotherapy for insomnia and the use of tapering strategies for benzodiazepines. Specific messages delivered are summarized in Table 1.

**Table 1:** Messages delivered during detailing session

	
**Phase I**	**Treatment-resistant Depression in the Primary Care Setting**
	· Formulate a treatment plan at the start of therapy
	· Prior to switching or augmenting an antidepressant consider a longer trial (12–14 weeks) at a therapeutic dose
	· Triiodothyronine (T3, cytomel) is an effective and well tolerated augmentation agent
	**Use of Atypical Antipsychotics in Treatment –resistant Depression**
	· Consider T3 (or lithium) prior to considering atypical antipsychotic augmentation agent
	· Atypical antipsychotics are associated with metabolic abnormalities and require regular monitoring
**Phase II**	**Pharmacotherapy options for Insomnia**
	· Cognitive behavior therapy and pharmacologic treatment approaches have similar effectiveness
	· All sedative/hypnotics appear to be comparable in treating insomnia
	· Clinical data regarding sedating antidepressants and antipsychotics are lacking
	**Clinical use and comparative effectiveness of benzodiazepines**
	· Long-term benzodiazepine use is rarely warranted
	· Withdrawal of benzodiazepine has led to improvements in cognitive functioning, balance, and memory without worsening insomnia (particularly in frail elderly)
	· Discontinuation should always include gradual tapering

The academic detailing was performed by a clinical pharmacist with specialty training in psychopharmacology (AH). Detailing material comprised a mix of different formats and media tailored to each clinic environment. The material was developed internally and selected from existing education available from reputable sources. The central therapeutic message for each interaction was incorporated into a short discussion, facilitated with a slide set. Two clinics received face-to-face visits and slides were printed out as handouts. Education delivered to the distance sites engaged with the detailer in differing ways. In one clinic, detailing was conducted using video conference. Information on slides was supplied beforehand as a handout as well as visually displayed during the discussion. For the other distance site, the education was delivered as a self-paced, Flash-enabled learning module accessed via the web, with the detailer’s contact information made available for follow-up questions or comments. Modules were developed using the software Articulate Presenter™. This software enables the addition of self-paced narration and interactivity within existing Windows PowerPoint slides. Although we did not directly track how many users completed each module we were able to monitor internet traffic through the website. In the year following introduction of the modules we were able to document 1289 views of the treatment resistant depression module, 787 views of the atypical antipsychotic module, 391 views of the insomnia module, and 309 views of the benzodiazepine module.

In all cases, a series of stand-alone handouts were developed that contained a summary of the key evidence supporting the clinical message for that visit. Several of these handouts contained information describing clinic-specific prescribing practices related to that message. We used pharmacy and medical claims from the state Medicaid program to create prescribing reports for three of the four modules. For the treatment-resistant depression module, our handout highlighted clinic and state level antidepressant market share, persistence with antidepressant therapy after initiation, and metrics describing the proportion of individuals who switched to a different antidepressant, or added an augmenting agent. The atypical antipsychotic utilization report also contained state and local market share data as well as information about metabolic testing (i.e. medical claims for glucose or lipid testing). A final utilization report focused on medications for insomnia and contained market share data as well as a synopsis of a recent systematic review. We also used existing clinician handouts produced through the AHRQ Effective Health Care program where applicable. All material was delivered in person or electronically to all 4 clinics during the outreach. Educational materials used for this program, including web modules, can be accessed through the ROAD website http://pharmacy.oregonstate.edu/drug_policy/road.

We assessed clinician satisfaction, perceptions of service usefulness, and willingness to continue with the program using a short, written survey delivered at the midpoint and project completion stages. This two-three page survey consisted of several multiple choice Likert-scale questions that asked clinicians to rate their impressions and satisfaction with various aspects of the service. Several open-ended questions were also used to gain additional insights into what was of specific value and what could be improved. Surveys were delivered via SurveyMonkey™. Chi-square tests of proportion were used to evaluate the statistical significance of survey responses between clinicians receiving face-to-face versus distance detailing. This study was approved by the Oregon Health & Science University IRB (#5012). Informed consent was obtained for all clinicians participating in survey assessments.

## Results

Table 2 describes participating clinic and community characteristics as well as the detailing outreach schedule. In addition to the initial focus group meeting, the ROAD team engaged with each clinic four times over an eight month period. While Clinics B and C were in the same community, Clinic B received face-to-face visits and Clinic C was detailed through video conference technology during a regularly scheduled noon conference period. In addition to the self-paced web modules, all printed materials prepared for in-person and video detailing were packaged electronically and sent via email to Clinic D. Staff at Clinic D were encouraged to ask questions or comments to the detailer through phone or email.

**Table 2:** Clinic descriptive information

	**In person**		**Distance**	
**Clinic**	**A**	**B**	**C**	**D**
Rurality (RUCA Category)*	10.3	4	4	7.4
	Isolated small rural town	Large rural city/town population 10,000-49,999	Large rural city/town population 10,000-49,999	Small rural town with flow to an urban cluster of 10,000-49,999
**Community Size**	414	20,840	20,870	4,154
**Clinic Patient Panel Size**	2648	12,647	8,000	5163
**Clinicians**	4	8	16	13
**Outreach Dates**				
Focus group	July 1, 2009	Aug 19, 2009	Aug 18, 2009	July 7, 2009
Period 1	Nov 13, 2009	Nov 18, 2009	Nov 12, 2009	Nov 25, 2009
	Jan 29, 2010	Jan 20, 2010	Jan 25, 2010	Feb 3, 2010
Period 2	May 15, 2010	May 19, 2010	May 4, 2010	June 3, 2010
	June 16, 2010	June 18, 2010	June 1, 2010	July 13, 2010

*Rurality as defined by the Rural–urban Commuting Areas (RUCA) developed by the University of Washington Rual Health Research Center (http://depts.washington.edu/uwruca/).

RUCA is a classification algorithm used by federal organizations and researchers to characterize rural and urban status of communities.

**Figure 1:**
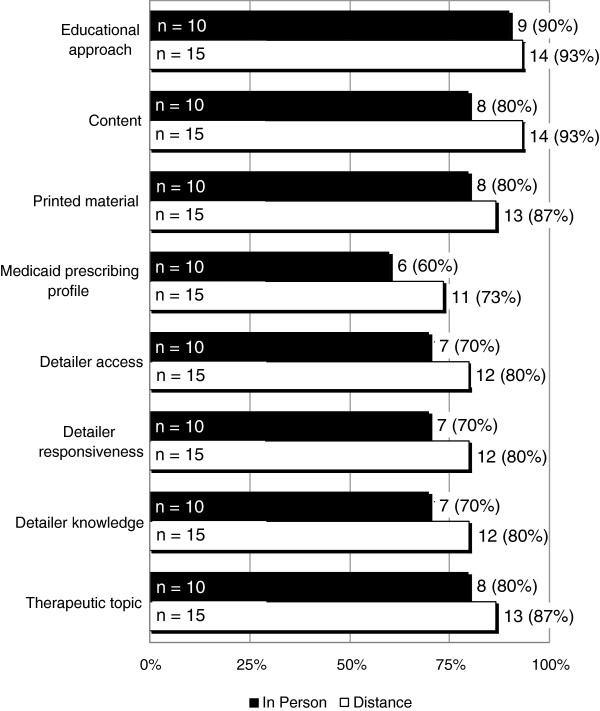
**Survey question: rate your satisfaction with the following ROAD service components on a scale of: very unsatisfied, unsatisfied, satisfied, or very satisfied. n represents number of respondents.** Numbers and percentages on each bar reflect those who responded satisfied or very satisfied.

Electronic surveys were sent out to clinicians at the midpoint period, and again following completion of the pilot. The response rates were 25/41 (61%) and 32/41 (78%) for the midpoint and final surveys respectively. Results are summarized by detailing type, i.e., in-person versus distance detailing. Figure 1 shows results when participants were asked about their satisfaction with components of the ROAD service rated on a four-point scale that included the following descriptions: *very unsatisfied, unsatisfied, satisfied,* or *very satisfied*. Of those who responded, 90% or more were *satisfied* or *very satisfied* with the overall educational approach used at their clinic. Between 70% and 90% of respondents indicated being *satisfied* or *very satisfied* with other components of the detailing service such as educational content, utility of the printed material, detailer access and responsiveness, knowledge of the detailer, and therapeutic topics. The Medicaid prescribing profile achieved the lowest satisfaction rating with only 60%-73% of respondents indicating they were *satisfied* or *very satisfied*. The differences between the in-person and distance satisfaction did not achieve statistical significance for any comparison.

**Figure 2:**
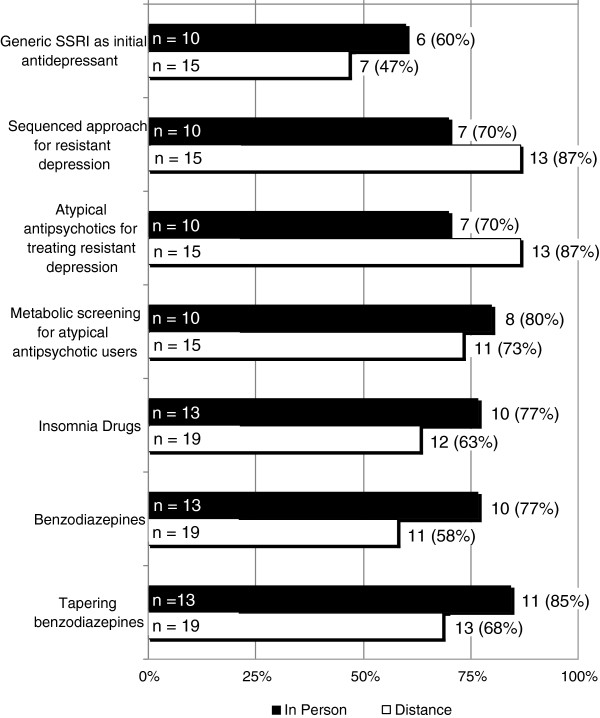
**Survey question: rate your perception of the impact of this education on how you practice on a scale of: no change, unlikely to change, likely to change, or have or will change. n represents number of respondents.** Numbers and percentages on each bar reflect those who responded likely to change or have or definitely will change.

Figure 2 illustrates responses to the question, “Rate your perception of the impact of this education on how you practice,” with choices ranging from *no change, unlikely to change, likely to change,* or *have or will change*. The first four questions in Figure 1 relate to topics covered during the first detailing period (selective serotonin reuptake inhibitor (SSRI) use, sequenced treatment selection, atypical antipsychotics, and metabolic screening). The last three topics were covered during the second detailing period (insomnia drugs, benzodiazepines, and benzodiazepine tapering). Responses are described as the proportion of individuals indicating they would *likely change* or *have or will change*. Generally, 70-80% of respondents indicated they would *likely change* or *have or will change* practices related to treatment-resistant depression and metabolic screening for atypical antipsychotic users. A lower proportion of respondents (47%-60%) indicated they would *likely change* or *have or will change* practices related to generic SSRIs as initial antidepressant use. Over 75% of respondents who received in-person education indicated they would *likely change* or *have or will change* their prescribing behavior related to drugs for insomnia and benzodiazepines. Among those receiving distance education 58% to 68% of respondents indicated a similar level of commitment to change respectively. The differences between groups did not reach statistical significance. For five of seven content areas, those receiving in person detailing reported a higher likelihood of changing their behavior compared to those in the distance detailing group.

**Figure 3:**
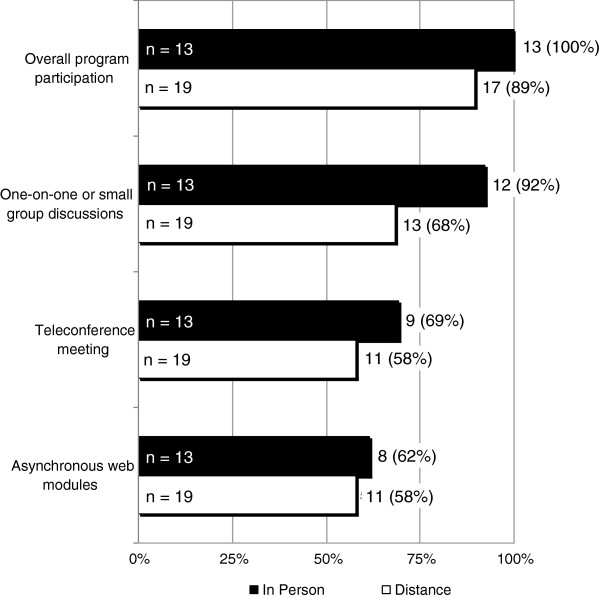
**Survey question: Rate the likelihood of participating in the following activities if this program is expanded in the future on a scale of: will not participate, unlikely to participate, likely to participate, will participate.** n represents number of respondents. Numbers and percentages on each bar reflect those who responded likely to participate or will participate.

Figure 3 describes the likelihood clinicians would participate in the program if it were expanded or continued in the future. In general, of those receiving in-person detailing, 100% indicated they *will* or *would likely participate* in the future. Overall, nearly 90% of those receiving distance-detailing indicated they *will* or *would likely participate* in the future. Participants were then asked about their likelihood of participating in specific formats of detailing. The proportion of individuals indicating they *will* or *would likely participate* declined to between 58% to 70% when the detailing involved distance approaches such as teleconference and web modules. None of the differences between detailing approaches reached statistical significance.

A summary of open-ended comments from clinicians about what they liked most about the ROAD project and what they would change for the future can be found online (http://pharmacy.oregonstate.edu/drug_policy/sites/default/files/ROAD/Documents/ROAD_appendixTable.pdf).

## Discussion

The ROAD pilot project of alternative approaches to delivering academic detailing was well received by participating family medicine clinics. Survey data overwhelmingly indicate clinicians were satisfied with the content and approaches used. The component with the highest reported satisfaction involved the specific content covered during the outreach. The component achieving the lowest level of reported satisfaction was our Medicaid prescribing profile. This finding is not entirely surprising. While we intended our prescribing reports to inform clinicians about their own current prescribing practices, the number of claims identified for each prescribing indicator was generally small and highly variable. Nevertheless, participating clinicians uniformly indicated they would likely or had already changed behavior related to several of the educational messages delivered. On average 60% to 75% of respondents indicated their willingness to change behavior. Clinicians indicated the least likelihood for changing the use of generic SSRI antidepressants as the initial choice for treating depression. This was unexpected because prevailing evidence shows only modest differences in effectiveness between commonly used SSRI antidepressants [17]. One possible explanation is that nearly 75% of current prescribing is already for generic SSRI antidepressants leaving little room for changes in practice to occur.

While satisfaction between the face-to-face and distance detailing approaches were similar, clinicians’ perceptions of impact seemed to favor the face-to-face encounter over the distance approach. Clinicians who received in–person outreach rated their likelihood of changing as qualitatively higher for 5 of 7 categories. While virtually all participants indicated their willingness to continue future participation in the program, enthusiasm for the outreach was diminished for distance approaches such as asynchronous web modules and teleconference. Unstructured responses solicited from the participants confirm the bias towards in-person detailing, with several individuals suggesting the teleconference approach was not effective. Many in the field strongly advocate that academic detailing requires a face-to-face interaction for the purpose of relationship building, establishing credibility, and facilitating two-way communication between the clinician and the detailer [4]. In theory, much of this communication could potentially be accomplished through video conferencing. In practice, however, it was our impression that barriers to open dialog still exist with such an approach, such as the diminished ability to recognize body language. Future programs might benefit from a hybrid approach where less frequent visitations are coupled with online or other distance technologies. Also, our program used an approach where clinicians engaged with the clinical pharmacist in small groups (versus one-on-one). We used this model because of the logistical difficulties anticipated with individual clinician meetings and because preliminary focus group suggested a preference for it. Although much of what has been described in the literature consists of a one-on-one detailing model, some evidence exists showing that small group detailing is an effective alternative to individual encounters [18].

Project resources allowed for only a small amount of clinical pharmacist (detailer) time to deliver education and to assist in content development. Content development and generation of provider prescribing profiles was resource-intensive, requiring significant time for research, programming and design. We question the ultimate return on investment for developed reports given the limitations and narrow population scope of the summarized data. In developing other educational tools, we attempted to leverage existing evidence-based products. For example, we used the clinician handout for treatment of depression developed by the AHRQ Effective Healthcare Program. We also explored the existence of materials on academic detailing websites in Canada, Australia, and the Harvard program which operates in Pennsylvania (http://www.rxfacts.org). Unfortunately, at the time there was a scarcity of material on many of the mental health topics we elected to detail on, such as treatment-resistant depression.

Our findings are tempered by several limitations. First, while the response rate from our survey was moderate, the overall response was low and likely not adequately powered to detect significant differences between the two detailing approaches. Initially, we explored using Medicaid pharmacy data to both generate prescribing profiles and to measure changes in behavior following our intervention. It became clear during the design of these profiles that the number of patients, and ultimately pharmacy claims, associated with each clinician was very small. As a result, our prescribing profiles were likely of little value. Similarly, the use of pharmacy claims to quantify changes in behavior was highly variable and unreliable.

Each clinic was given a $1000 honorarium for participating in the ROAD project. Our intent was to provide clinics with a gesture of gratitude for time spent with research-related activities, such as filling out surveys and participating in the focus group, rather than as an incentive to receive the detailing itself. It is unclear if clinics would be as willing to participate in future efforts if the financial reimbursement was eliminated. While Medicaid data limitations curtailed our ability to document changes in prescribing behavior, the outreach was well received and helpful to clinicians. In the near future, the State of Oregon is rolling out an all–payer, all-claims dataset that will serve as a repository for medical and pharmacy claims for private (group and self-insured) and public (Medicaid and Medicare) insured individuals (~3 million covered lives per year) in the state. The implementation of Oregon’s all–payer, all-claims dataset is a promising development that could assist in generating more accurate and comprehensive prescribing profiles, targeting specific providers, and enhance our ability to evaluate future programs.

While many academic medical centers have taken steps to curtail influence of the pharmaceutical industry on the continuing education of their clinicians, family physicians in small independent practices may continue to rely on industry for prescribing information as well as the provision of drug samples [10,19]. The Institute of Medicine’s consensus report entitled “Conflict of Interest in Medical Research, Education, and Practice” published in 2009 recognizes the information needs of clinicians and proposes that academic detailing programs may be an attractive alternative for clinicians to obtain non-commercial prescribing information [20]. In the US, academic detailing activities have primarily been localized state programs, although AHRQ is currently funding a national effort focused on dissemination and translation of its Effective Health Care products to primary care clinicians [21]. Several lessons emerge from this project that will assist in informing a longer-term more sustained effort in the state. First and foremost, the distance approach, as formulated in this project, was not viewed as favorably as outreach delivered in person. This is consistent with the theoretical framework of detailing which stresses the importance of relationship building and credibility on affecting change [4,22]. Future work should either abandon this approach or supplement it with individual interaction. At the very least, if a similar approach is used, it will be important to actively assess and incentivize provider engagement, as well as knowledge attainment and retention with the distance modules. Video conferencing may still be a viable option if it can be configured in a way that facilitates the personal connection between the detailer and provider. Also, it is unclear at what interval or intensity of engagement needs to occur in order to achieve a desired change. Understanding this will be critical for sustaining programs that cover geographically distant areas. Finally, the effort required to synthesize evidence into usable clinical practice recommendations it not a trivial matter. Although a variety of organizations have built a sizable library of material that can be used in academic detailing efforts, gaps remain. The work of packaging evidence syntheses for clinical use will grow substantially as evidence from the US comparative effectiveness research enterprise accumulates.

## Conclusion

The goal of this pilot study was to explore the feasibility and satisfaction associated with a distance learning approach to academic detailing. While study limitations and sample size prohibit definitive conclusions, our data suggest participants favored, and were generally more responsive to, in-person approaches compared to distance interactions. Future efforts will be directed at evaluating the clinical and economic benefits of detailing in rural family practice settings.

## Abbreviations

AHRQ: Agency for Healthcare Research and Quality; OHSU: Oregon Health & Science University (OHSU); ORPRN: Oregon Rural Practice-based Research Network (ORPRN); ROAD: Rural Oregon Academic Detailing Project; SSRI: Selective serotonin reuptake inhibitor; STARD: Sequenced Treatment Alternatives to Relieve Depression.

## Competing interest

None of the authors reports any conflicts of interest.

## Authors’ contributions

DMH: Secured funding, concept, design, analysis, drafting and critical revision of manuscript. AH: drafting and critical revision of manuscript. LM: Analysis, drafting and critical revision of manuscript. DGH: drafting and critical revision of manuscript. LF: Recruited clinics, design, analysis, drafting and critical revision of manuscript. All authors read and approved the final manuscript.

## Pre-publication history

The pre-publication history for this paper can be accessed here:

http://www.biomedcentral.com/1471-2296/13/129/prepub
